# Novel chemotherapeutic agent, FND-4b, activates AMPK and inhibits colorectal cancer cell proliferation

**DOI:** 10.1371/journal.pone.0224253

**Published:** 2019-10-24

**Authors:** Heather F. Sinner, Jeremy Johnson, Piotr G. Rychahou, David S. Watt, Yekaterina Y. Zaytseva, Chunming Liu, B. Mark Evers

**Affiliations:** 1 Department of Surgery, University of Kentucky, Lexington, Kentucky, United States of America; 2 Markey Cancer Center, University of Kentucky, Lexington, Kentucky, United States of America; 3 Department of Toxicology and Cancer Biology, University of Kentucky, Lexington, Kentucky, United States of America; 4 Department of Molecular and Cellular Biochemistry, University of Kentucky, Lexington, Kentucky, United States of America; 5 Center for Molecular Medicine, Organic Synthesis Core, University of Kentucky, Lexington, Kentucky, United States of America; University of Navarra, SPAIN

## Abstract

Colorectal cancer (CRC) is the second leading cause of cancer deaths in the US with the majority of deaths due to metastatic disease. Current chemotherapeutic regimens involve highly toxic agents, which limits their utility; therefore, more effective and less toxic agents are required to see a reduction in CRC mortality. Novel fluorinated N,N’-diarylureas (FND) were developed and characterized by our group as potent activators of adenosine monophosphate-activated kinase (AMPK) that inhibit cell cycle progression. The purpose of this study was to determine the effect of a lead FND compound, FND-4b, either alone or combined with PI-103 (a dual PI3K/mTOR inhibitor) or SN-38 (active metabolite of irinotecan) on cell cycle arrest and apoptosis of CRC cell lines (both commercially-available and novel lines established from our patient population). Treatment with FND-4b for 24h resulted in a marked induction of phosphorylated AMPK expression and a concomitant reduction in markers of cell proliferation, such as cyclin D1, in all CRC cell lines. Apoptosis was also notably increased in CRC cells treated with FND-4b. Regardless of the genetic profile of the CRC cells, FND-4b treatment alone resulted in decreased cell proliferation. Moreover, the combination of FND-4b with PI-103 resulted in increased cell death in all cell lines, while the combination of FND-4b with SN-38 resulted in increased cell death in select cell lines. Our findings identify FND-4b, which activates AMPK at micromolar concentrations, as a novel and effective inhibitor of CRC growth either alone or in combination with PI-103 and SN-38.

## Introduction

Colorectal cancer (CRC) is the second leading cause of cancer deaths in the United States [1, 2]. A multimodal approach to treatment is necessary to cure CRC and includes both surgical resection as well as systemic chemotherapy. The first-line systemic therapy for CRC is comprised of a fluoropyrimidine (5-FU) used in various combinations and schedules with leucovorin, irinotecan, or oxaliplatin [3]. Despite advances in cytotoxic and targeted therapy, drug resistance (intrinsic or acquired) remains a great challenge and is considered to be a major cause for treatment failure in cancer [4].

Deregulation of cellular metabolism and cell proliferation is a major mechanism of tumor cells. When cells are metabolically stressed, the intracellular ratio of adenosine monophosphate (AMP) to adenosine triphosphate (ATP) is increased, which in turn, activates AMP-activated protein kinases (AMPKs). AMPK activation then regulates various cellular processes, such as cell proliferation, cell polarity, autophagy, and apoptosis [5, 6]. Specifically, activation of AMPK inhibits cell growth by engaging p53-dependent cell cycle arrest and downregulation of mTORC1 activity, while a lack of AMPK signaling impairs autophagy and apoptosis [7]. Neoplastic tissue make effective use of this regulatory mechanism in order to sustain unregulated growth by down-regulating AMPK signaling. As such, AMPK activators represent a potential target for tumor suppression. Among the AMPK activators currently studied are the anti-diabetic drug metformin and 5-amino-1-β-D-ribofuranosyl-imidazole-4-carboxamide (AICAR), which have been shown to reduce the risk of colorectal cancer, especially in diabetic patients [8]. However, both of these drugs have failed to inhibit tumor growth in certain CRC cell lines (e.g., HCT116 wild-type p53) [5, 9]. Thus, further research into novel AMPK activators is needed to identify an AMPK activator that comprehensively inhibits cancer cell growth and tumorigenesis, despite the mutation profile of the tumor.

Novel fluorinated N,N’-diarylureas (FNDs) were developed and characterized by our group as potent activators of AMPK that inhibit cell cycle progression [10]. These FNDs structurally resemble the multikinase inhibitors, regorafenib and sorafenib, which are approved for the treatment of colon cancer, renal cancer, and advanced liver cancer [11, 12]. Previously, we reported the ability of eight FND compounds to inhibit growth and induce apoptosis in CRC stem cell lines and showed that a lead FND compound, FND-4b, had similar effects as metformin on cell cycle inhibition [13]. Importantly, the effect of FND-4b on cell cycle inhibition was noted at 20μM, as compared to the 10,000μM dose of metformin required to achieve similar results.

To better characterize the pharmacologic potential of FND-4b as a novel chemotherapeutic agent, we investigated the effect of FND-4b, either alone or in combination with PI-103, a dual inhibitor of Class IA phosphatidylinositide 3-kinase (PI3K) and mTOR [14–18], or SN-38, the active metabolite of the topoisomerase inhibitor irinotecan [19], on cell cycle arrest and apoptosis of commercially-available human CRC cell lines. We then expanded our study to include primary CRC cell lines established from patient-derived xenografts (PDXs) in order to provide further evidence of FND-4b as an effective tumor suppressor in CRCs with a variety of mutation profiles. Our study identifies FND-4b as a novel and effective AMPK activator that inhibits CRC growth when used alone or in combination with other therapeutic agents.

## Materials and methods

### Treatment compounds and antibodies

FND-4b was synthesized as previously described.[10] PI-103 (#S1038) and SN-38 (#S4908) were obtained from Selleck Chemicals (Houston, Texas). See chemical structures in S1 Table. Each compound was dissolved in dimethyl sulfoxide (DMSO). Diluted stock solutions, in DMSO, were stored at -20°C.

Antibodies for western blot analysis included the following: PARP (Cell Signaling #9542, 1:1000), p-AMPKα (Cell Signaling #2535, Thr172, 1:1000), cyclin D1 (Abcam AB134175, 1:2500); p-AKT (Cell Signaling #4060, 1:1000); AKT (Cell Signaling #2920, 1:1000); β-actin (Sigma Aldrich #A5441, 1:10,000); anti-rabbit and anti-mouse (Santa Cruz Biotechnology, #SC-2054 and #SC-2055, 1:10,000). Activation of the mTORC signaling pathway was evaluated by expression of p-AKT and p-AMPKα. Cell proliferation was evaluated by cyclin D1 expression. Apoptosis was evaluated by PARP cleavage.

### CRC cell lines and culture maintenance

#### Commercially-available CRC cell lines

The human CRC cell lines (HT29, HCT116- p53 wild type, and LS174T) were obtained from ATCC (Manassas, Virginia) and authenticated in February 2016 (Genetica DNA Laboratories, Cincinnati, OH). DLD1 PI3KCA mutant cells were obtained from ATCC, while DLD1 PI3KCA wild type cells were a gift from Dr. Jing Wang (The University at Buffalo-SUNY). Genetic mutations with potential implication for treatment resistance to one of the studied drug agents include a PI3K mutation in all four main cell lines tested (S2 and S3 Tables). HT29, HCT116, and LS174T cells were grown in McCoy’s 5A media (Sigma Aldrich, St. Louis, Missouri) containing 10% fetal bovine serum (FBS) and 1X antibiotic-antimycotic (Life Technologies, Carlsbad, California) and cultured at 37°C under an atmosphere containing 5% CO_2_. DLD1 mutant and wild type cells were grown in RPMI-1640 medium (Gibco, Thermo Fisher Scientific, Waltham, Massachusetts) containing 10% FBS and 1X penicillin-streptomycin and cultured at 37°C and 5% CO_2_. At the time of experimentation, cells were in a passage range of 15–20 and cells were seeded at 8x10^5^ cells per well.

#### PDX-derived CRC cell lines

This research utilized human CRC tissues collected from consented patients who had undergone surgical resection at UK Medical Center and was approved by the UK Institutional Review Board (IRB # 16-0439-P2H). Mice used in this research were housed in the UK Division of Laboratory Animal Resources, which is fully accredited by the Association for the Assessment and Accreditation of Laboratory Animal Care. All procedures involving mice were prospectively approved by and performed under supervision of the University of Kentucky’s Institutional Animal Care and Use Committee (IACUC #2016–2418; IRB #44068) and according to regulations stipulated by the Animal Welfare Act and the Guide for the Care and Use of Laboratory Animals. Euthanasia was conducted using the CO_2_ chamber or decapitation, consistent with the 2000 Report of the American Veterinary Medical Association panel.

Human CRC tissues were collected after surgical resection and implanted into NOD *scid* gamma mice (NSG^™^) (The Jackson Laboratory, Bar Harbor, ME) to establish the PDX. The resultant primary CRC cell lines (Pt.93, Pt.130) were established after three sequential generations in mice and authenticated as unique human cell lines in February 2016 (Genetica DNA Laboratories). After this, we established two other primary CRC cell lines derived from PDX tumors (Pt.2377-Primary Tumor (1°), Pt.2377-Liver Metastasis (LM)) in the same manner and used Next Generation Sequencing to compare the genetic profile of 198 oncogenes to original patient tumors (University of Kentucky Oncogenomics Core). Genetic mutations with potential implication for treatment resistance to one of the studied drug agents include a PI3K mutation in Pt.2377-1° and Pt.2377-LM (S2 and S3 Tables). All cell lines were grown in DMEM-high glucose media (Sigma Aldrich) containing 10% FBS and 1X antibiotic-antimycotic (Life Technologies). At the time of experimentation, cells were in a passage range of 5–10 and cells were seeded at 1x10^6^ cells per well.

### Western blot analysis

Total protein lysates were resolved on a 4%–12% Bis-Tris gel and transferred to Immobilon PVDF transfer membranes. Membranes were incubated for 1h at room temperature in blocking solution (TRIS-buffered saline containing 10% nonfat dried milk and 0.1% Tween-20), followed by an overnight incubation in primary antibodies at 4°C. Membranes were washed in TBST and incubated with horseradish peroxidase-conjugated secondary antibodies for 30 min. After two additional washes, immune complexes on the membrane were visualized using Immobilon Western Chemiluminescent HRP substrate (EMD Millipore, Billerica, Massachusetts) or Amersham ECL (GE Life Sciences, Pittsburgh, Pennsylvania). Activation of the mTORC signaling pathway was evaluated by expression of p-AKT and p-AMPKα. Cell proliferation was evaluated by cyclin D1 expression. Apoptosis was evaluated by PARP cleavage.

### Cell proliferation assay

Cells were seeded in 96-well plates in antibiotic-free media 24h prior to treatment (commercially available cell lines at 5x10^3^ cells/well, PDX-derived cell lines at 8x10^3^ cells/well; 100 μL). After treatment with respective drug or combination of drugs, cell proliferation was evaluated at 48h treatment duration (commercial cell lines) or 144h duration (PDX-derived cell lines) using the CytoScan^™^ SRB Cytotoxicity Assay (G-Biosciences^®^, USA) according to the manufacturer’s protocol. This assay utilizes sulforhodamine B (SRB) to bind to viable cellular protein in order to directly measure the total protein mass, which correlates to viable cell number. Cell viability was plotted as a percentage relative to the untreated control (media alone).

### DNA fragmentation assay

Cells were plated in 96-well plates 24h prior to treatment (commercially available cell lines at 5x10^3^ cells/well, PDX-derived cell lines at 8x10^3^ cells/well; 100 μL) and then treated with test drugs alone or in combination for 48h. DNA fragmentation was evaluated by examination of cytoplasmic histone-associated-DNA-fragments after induced cell death using the Cell Death Detection ELISA^PLUS^ Kit (Version 15, Roche Molecular Biochemicals, Mannheim, Germany) according to the manufacturer’s protocol. DNA fragmentation was plotted as absorbance (mU).

### Statistical analysis

Analysis of variance was employed for comparison of cell proliferation and cell death across various treatment combinations of FND-4b, PI-103, and SN-38. Adjusted p-values using the Holm-stimulated method were calculated for pairwise comparison of each drug treatment with control and with each other drug. Where applicable, results are expressed as a mean ± standard deviation (SD). Significance was pre-determined at p<0.05.

## Results

### AMPK activation by FND-4b is dose- and time-dependent

**Fig 1:**
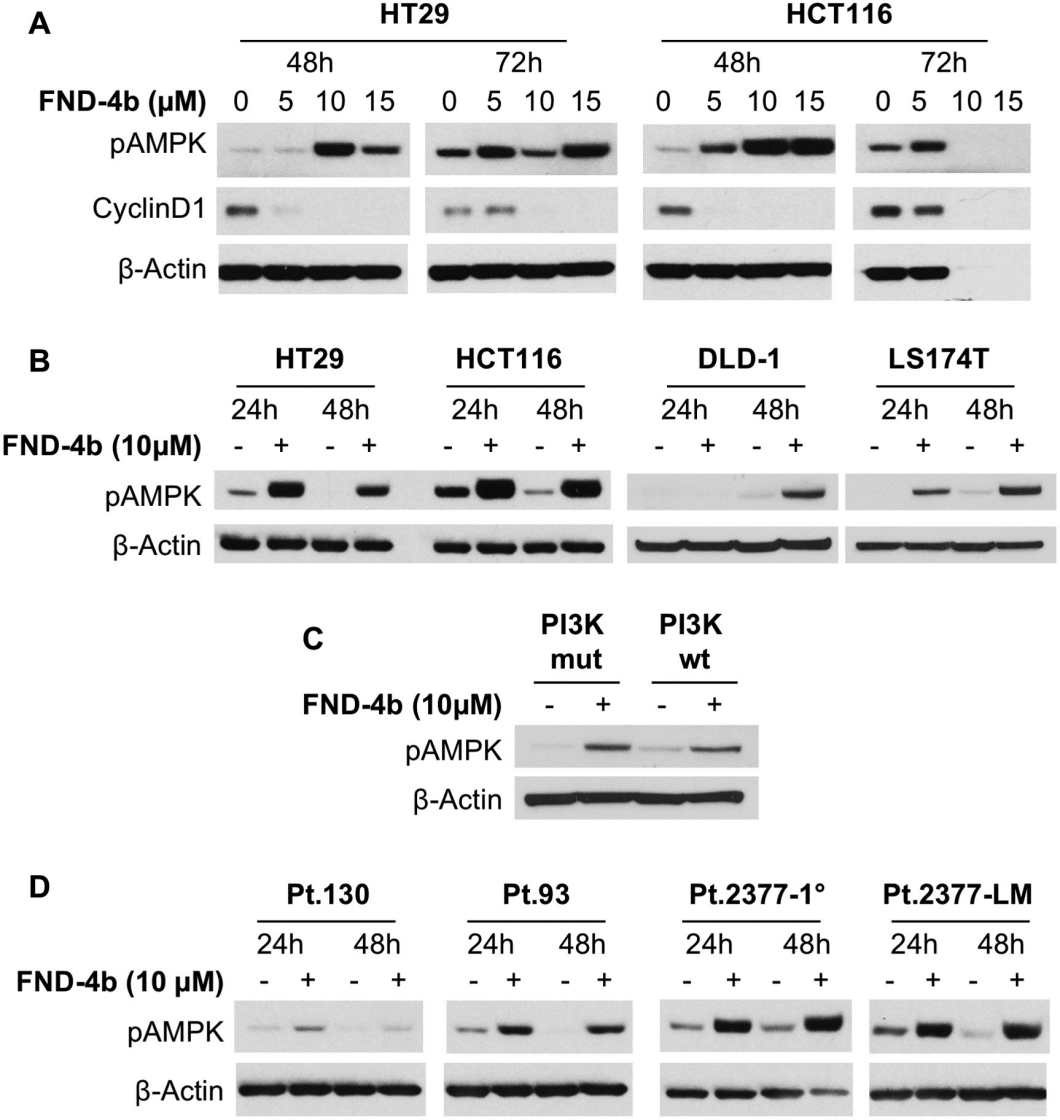
Determining effective treatment dose and duration for commercially-available and PDX-derived colorectal cell lines. (A) Treatment of HT29 and HCT116 cells with FND-4b at varying concentrations for 48h and 72h demonstrated maximal AMPK activation with 10μM FND-4b at 48h. (B) Treatment of HT29, HCT116, LS174T, and DLD1 with 10μM FND-4b for 24h or 48h duration indicated optimal pAMPKα activity at 24h for all cells except DLD1. (C) Treatment of PI3KCA mut/wt DLD1 cells with 10μM FND-4b for 48 h resulted in similar levels of AMPK activation. (D) Treatment with 10μM FND-4b resulted in equivalent AMPK activation at 24h and 48h duration in all four PDX-derived CRC cell lines. Lowest effective treatment dose was 10μM FND-4b for 24h duration. β-actin was used as the loading control for all blots. The images are representative of three independent experiments.

To determine the effective *in vitro* concentration and duration for FND-4b treatment, commercially-available CRC cell lines (HCT116 and HT29) were treated with FND-4b at different dosages (0, 5, 10, 15μM) for 48 h and 72 h (Fig 1A). We found that 10μM FND-4b effectively increased pAMPK expression and decreased cyclin D1 expression in HT29 and HCT116 cells (Fig 1A). We then compared the degree of AMPK activation at 24 h with that at 48 h and expanded our analysis to include two other commercially-available cell lines (LS174T and DLD1) (Fig 1B). We found that maximal AMPK activation occurred at 24h after treatment with 10μM FND-4b in all cell lines except for DLD1, which had maximal activation after 48 h (Fig 1B). Since these four cell lines have PI3KCA mutations (mut), we also incorporated a modified wild type (wt) PI3KCA DLD1 cell line into our analysis (Fig 1C). Treatment of DLD1 mut/wt cells with FND-4b for 48 h yielded similar levels of AMPK activation, indicating that FND-4b signaling is independent of the PI3K pathway (Fig 1C). To determine if a similar FND-4b treatment dosage and duration was effective in the PDX-derived CRC cell lines (Pt.130, Pt.93, Pt.2377-1°, Pt.2377-LM), we treated these cells with 10μM of FND-4b for both 24h and 48h duration. Similar to the above experiments, the optimal FND-4b dosage and treatment duration were noted to be 10μM and 24h, respectively (Fig 1D); therefore, we utilized this dosage and treatment duration for the remainder of the *in vitro* experiments.

### Low-dose FND-4b treatment induces pAMPK expression in all CRC cell lines

**Fig 2:**
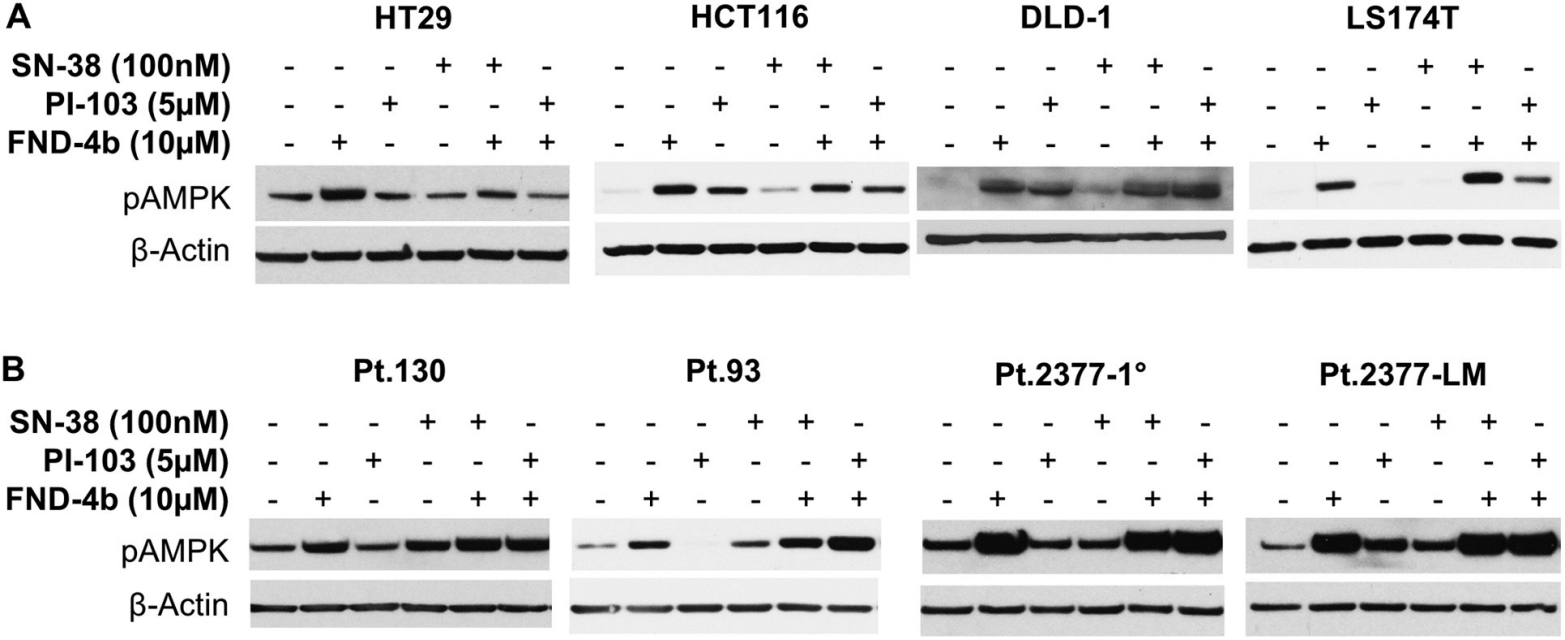
AMPK activation in commercial & PDX-derived CRC cell lines treatment combinations. Western blot analysis indicated that AMPK activation was increased in commercially-available CRC cell lines (A) and PDX-derived cell lines (B) after monotreatment or combination treatment with FND-4b (10μM), PI-103 (5μM), or SN-38 (100nM) for 24 h compared to the untreated control. β-actin was used as the loading control. The images are representative of three independent experiments.

To demonstrate the effectiveness of FND-4b treatment in the activation of AMPK, we treated each of the eight CRC cell lines with 10μM FND-4b alone or in combination with either 5μM PI-103 or 100nM SN-38 for 24h. We found that expression of pAMPK was increased, as compared to the untreated control, in all cell lines studied regardless of genetic mutation profile (Fig 2). Monotherapy with SN-38 or PI-103 had cell line-dependent effects on AMPK activation in both commercially-available and PDX-derived CRC cells. Of all cell lines tested, HT-29 cells were unique because the FND-4b-induced AMPK activation returned to its basal level in combination treatment with PI-103 or SN-38 (Fig 2A). Overall, the greatest expression of pAMPK was achieved with treatment of FND-4b alone or in combination with either PI-103 or SN-38, as compared to treatment with these drugs alone, indicating that FND-4b more efficiently targets AMPK activation.

### FND-4b treatment decreases proliferation of commercially-available CRC cells

**Fig 3:**
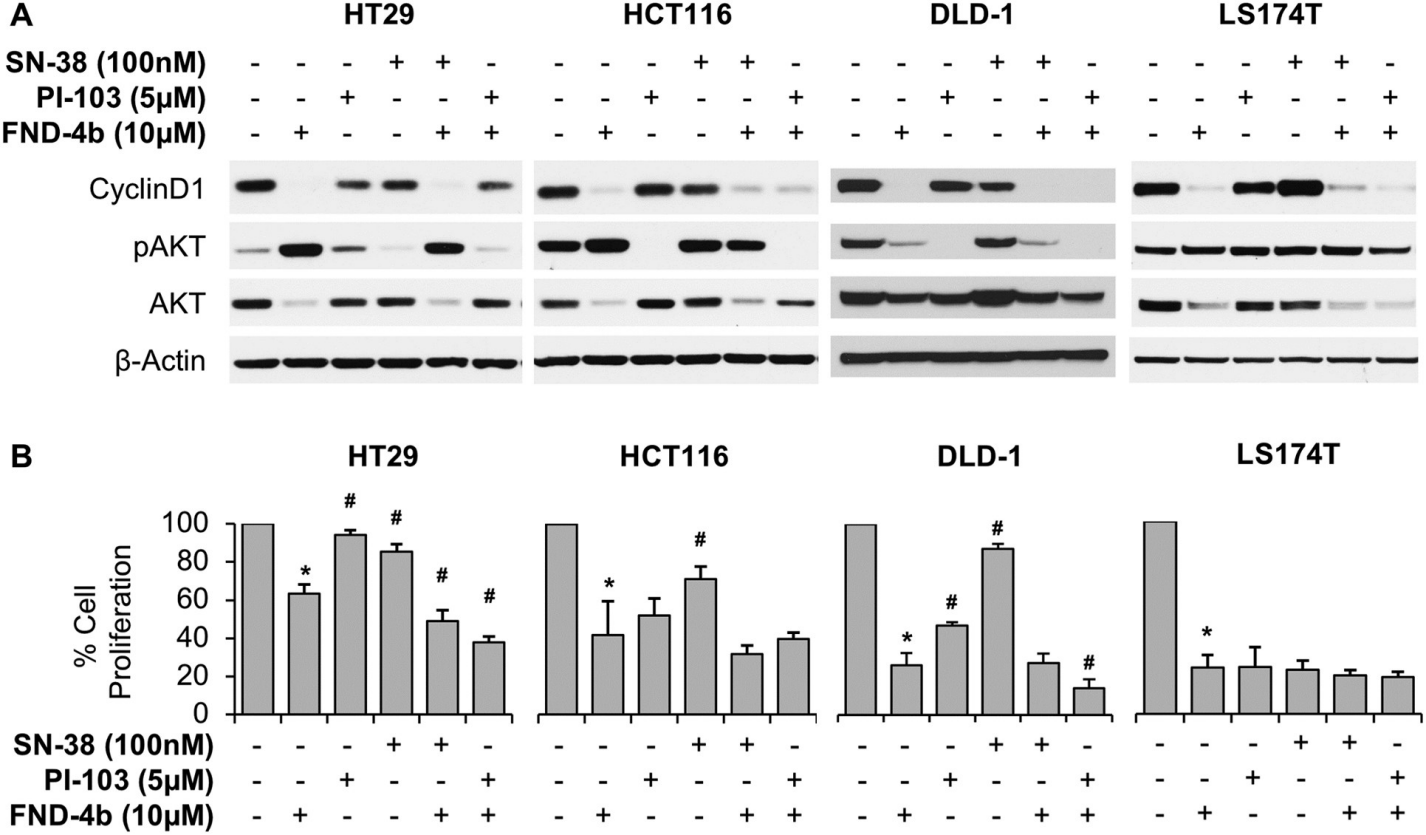
Cell proliferation of commercial CRC cell lines treatment combinations. (A) Western blot analysis of commercially-available CRC cell lines treated with 10μM FND-4b, 5μM PI-103, and 100nM SN-38, alone and in combination versus untreated control (media alone) for 24h duration. β-actin was used as a loading control. The images are representative of three independent experiments. In all CRC cell lines, FND-4b mono- and dual-treatment resulted in decreased cyclin D1 expression and—with the exception of DLD1—increased pAKT expression compared to untreated control. (B) SRB Cytotoxicity Assays of commercially-available CRC cell lines treated with 10μM FND-4b, 5μM PI-103, and 100nM SN-38, alone and in combination for 48h duration. Graphic representations are the mean ± SD plotted as a percentage relative to untreated control; each measurement was performed with 5 replicates. *p<0.0001 vs. control and #p<0.001 vs. FND-4b alone.

To determine the impact of treatments on CRC cell cycle progression and proliferation, we treated each CRC cell line with 10μM FND-4b, 5μM PI-103 and 100nM SN-38 as mono- or dual-therapy regimens for 24h and analyzed the protein expression of cyclin D1 by western blot. In all four commercially-available cell lines, we noted a dramatic decline in cyclin D1 expression following mono- or dual treatment with FND-4b, which was not demonstrated in the cells treated with PI-103 or SN-38 alone (Fig 3A).

In addition, we probed for other markers of cell cycle activity, including pAKT, which is expressed in the setting of ongoing cell growth and is inhibited by PI3K-inhibitors such as PI-103. Treatment with FND-4b alone or combined with PI-103, led to an increase in the expression of pAKT in both HCT116 and HT29 cell lines (Fig 3A). Similarly, in LS174T cells treated with FND-4b, we noted an increase in the ratio of pAKT to AKT. In DLD1 cells, on the other hand, FND-4b treatment caused a decrease pAkt expression. The accumulation of activated upstream peptides in the PI3K/mTOR signaling pathway in most cell lines further indicates that FND-4b acts on a target downstream of AKT and is thus not hindered by upstream mutations in the PI3K signaling pathway. This is important to note since many CRCs are resistant to current treatments due to PI3K mutations.

To evaluate the effect of FND-4b treatment on CRC cell survival, CRC cell lines were treated with FND-4b alone or in combination with PI-103 or SN-38 for 48h and analyzed by the CytoScan SRB Cytotoxicity assay. All cell lines demonstrated significant reduction in cell proliferation when treated by FND-4b, as compared to untreated control (p<0.0001 for each), which supports our western blot findings (Fig 3). HT29 cells treated with dual FND-4b + PI-103 therapy exhibited even greater reduction in cell proliferation, compared to FND-4b treatment alone (p<0.0001) (Fig 3B). Similarly, when HT29 cells were treated with the combination of FND-4b and SN-38, there was greater inhibition of cell proliferation compared to treatment with FND-4b alone (p<0.0001). We did not see additional cell cycle inhibition in HCT116 or LS174T cells treated with either FND-4b + PI-103 or FND-4b + SN-38 compared to FND-4b treatment alone, which indicates maximal AMPK activation in these cell lines by the FND-4b treatment, such that the addition of another agent provided no additional benefit. DLD1 cells treated with FND-4b + PI-103 had further cell cycle inhibition than FND-4b alone (p<0.001), but there was no additional impact when FND-4b was combined with SN-38. Taken together, these findings indicate that FND-4b has greater cytostatic properties than either PI-103 or SN-38 and show that FND-4b is effective in CRC cells that come from a variety of different consensus molecular subtypes (CMS).[20]

### FND-4b induces apoptosis in the commercially-available CRC cells

**Fig 4:**
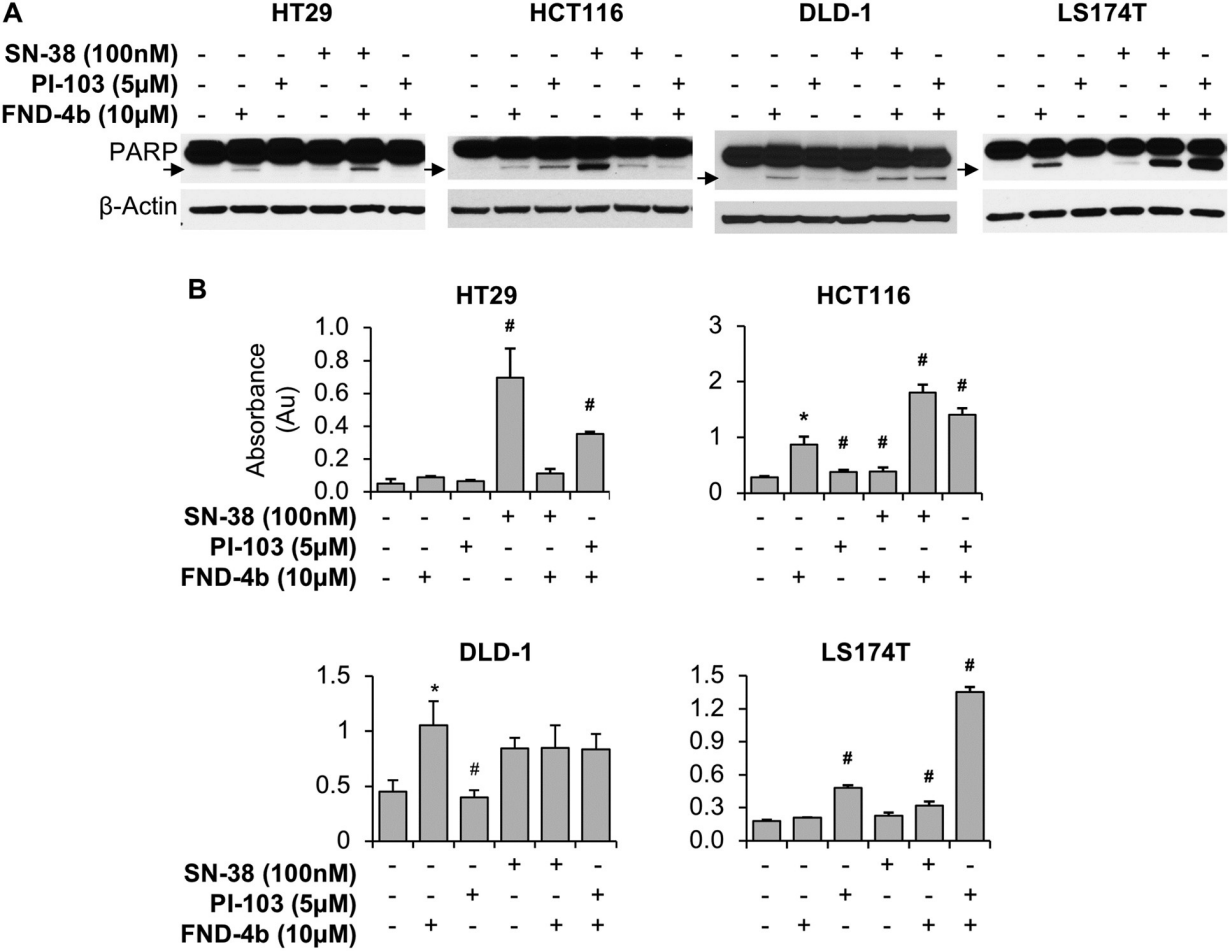
Apoptosis of commercial CRC cell lines treatment combinations. (A) Western blot analysis of commercially-available CRC cell lines treated with 10μM FND-4b, 5μM PI-103, and 100nM SN-38, alone and in combination versus untreated control (media alone) for 24h duration. PARP cleavage is indicated by an arrow; β-actin was used as a loading control. The images are representative of three independent experiments. In all CRC cell lines, PARP cleavage was increased as a result of FND-4b treatment compared to untreated control. (B) DNA fragmentation measured by Cell Death ELISA Assays of commercially-available CRC cell lines treated with 10μM FND-4b, 5μM PI-103, and 100nM SN-38, alone and in combination for 48h duration. Graphic representations are the mean ± SD of DNA fragmentation plotted as absorbance (Au); each measurement was performed with 3 replicates. *p<0.01 vs. control and #p<0.01 vs. FND-4b alone.

To determine the impact of FND-4b treatment on CRC apoptosis, we first probed western blots for PARP cleavage. In all cell lines, we found increased PARP cleavage in cells treated with FND-4b compared with the untreated control (Fig 4A). In HCT116 and HT29, the greatest increase in PARP cleavage was noted after treatment with SN-38 alone or combined with FND-4b. In contrast, in LS174T cells, the greatest PARP cleavage was noted after treatment with FND-4b in combination with PI-103. Similarly, cleaved PARP levels were highest in DLD1 cells after FND-4b was combined with either PI-103 or SN-38.

Next, we quantitated DNA fragmentation in each cell line 48h after drug treatment as a measure of apoptosis. Interestingly, in the HCT116 cell line, DNA fragmentation was more pronounced following treatment with FND-4b alone or in combination with either PI-103 or SN-38, compared to control treatment or PI-103 and SN-38 as single agents (Fig 4B). More specifically, the dual treatment with FND-4b + SN-38 led to significantly more apoptosis than any other treatment regimen (p = 0.002, as compared to the second highest amount of DNA fragmentation in the FND-4b + PI-103 group). Overall, HT29 and LS174T cell lines demonstrated DNA fragmentation to a lesser degree than HCT116, regardless of the treatment regimen suggesting that the effects of the drug treatments on HT29 and LS174T cells were more cytostatic compared with a more pronounced cytotoxic effect in HCT116 cells. DLD1 cells were unique because combination therapy with SN-38 or PI-103 did not cause more DNA fragmentation than treatment with FND-4b alone. In all cells except DLD1, the combination treatment of FND-4b with PI-103 resulted in a significant increase in apoptosis compared to treatment with FND-4b alone, indicating that the cytotoxic properties of FND-4b were enhanced by the addition of PI-103 treatment (comparison of FND-4b monotherapy to FND-4b + PI-103 combination therapy, HCT116 p = 0.0002, HT29 p = 0.005, LS174T p<0.0001), which was noted despite the mutation of PI3K in all three cell lines.

### FND-4b causes cell cycle arrest despite a variety of genetic mutations in PDX-derived CRC cell lines

**Fig 5:**
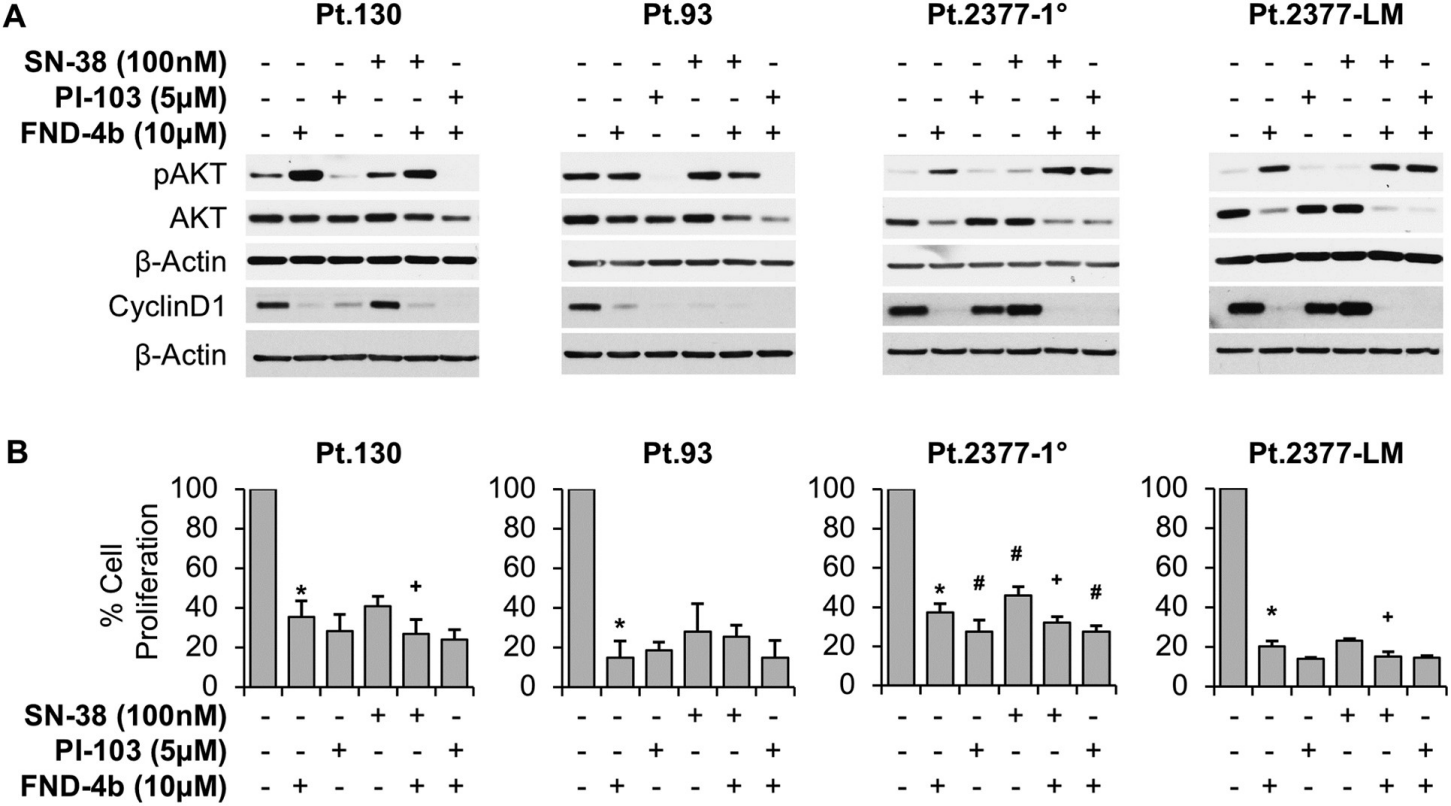
Cell proliferation of PDX-derived CRC cell lines treatment combinations. (A) Western blot analysis of PDX-derived CRC cell lines treated with 10μM FND-4b, 5μM PI-103, and 100nM SN-38, alone and in combination versus untreated control (media alone) for 24h duration. β-actin was used as a loading control. The images are representative of three independent experiments. In all CRC cell lines mono- or dual-treatment with FND-4b resulted in decreased cyclin D1 expression. (B) SRB Cytotoxicity Assays of PDX-derived CRC cell lines treated with 10μM FND-4b, 5μM PI-103, and 100nM SN-38, alone and in combination for 144h duration. Graphic representations are the mean ± SD plotted as a percentage relative to untreated control; each measurement was performed with 5 replicates. *p<0.0001 vs. control, #p<0.01 vs. FND-4b alone, and +p<0.05 vs. SN-38 alone.

To extend our findings regarding the effectiveness of FND-4b treatment on CRC cell growth and cell death, we next utilized four PDX-derived CRC cell lines, which were established from resected colon cancers at the University of Kentucky. In all four PDX-derived CRC cell lines, treatment with FND-4b, alone or in combination, caused almost complete inhibition of cyclin D1 expression (Fig 5A). Pt.93 appeared to be most susceptible to cell cycle inhibition with all 3 agents causing a significant reduction in cyclin D1 expression, while Pt.2377-1° and Pt.2377-LM only demonstrated inhibition of cyclin D1 expression when the treatment regimen included FND-4b. In addition, there was a notable increase in pAKT expression in the cell lines with PI3K mutations (Pt.2377-1° and Pt.2377-LM) following treatment with FND-4b or FND-4b in combination with PI-103 or SN-38. This was not noted in Pt.130 or Pt.93, which possess wildtype PI3K, thus indicating that PI-103 treatment inhibits pAKT expression only in susceptible cell lines.

To next evaluate the effect of FND-4b treatment on cell cycle arrest, PDX-derived CRC cell lines were treated with FND-4b alone or in combination with PI-103 or SN-38 for 144h and analyzed by CytoScan SRB Cytotoxicity assay (Fig 5B). There was a significant decrease in cell proliferation in Pt.130, Pt.2377-1°, and Pt.2377-LM cell lines (p = 0.02, p<0.0001, and p = 0.04, respectively) when these cells were treated with a combination of FND-4b and SN-38, as compared to SN-38 monotherapy. However, when comparing this combination therapy (FND-4b + SN-38) to treatment with FND-4b alone, there was no difference in cell proliferation in any of the PDX-derived cell lines (p>0.05 for each). This finding indicates that there is no additional cytostatic benefit to utilization of a dual treatment regimen of FND-4b + SN-38 compared to FND-4b treatment alone.

### FND-4b treatment increases apoptosis of PDX-derived CRC cells either alone or in combination with PI-103

**Fig 6:**
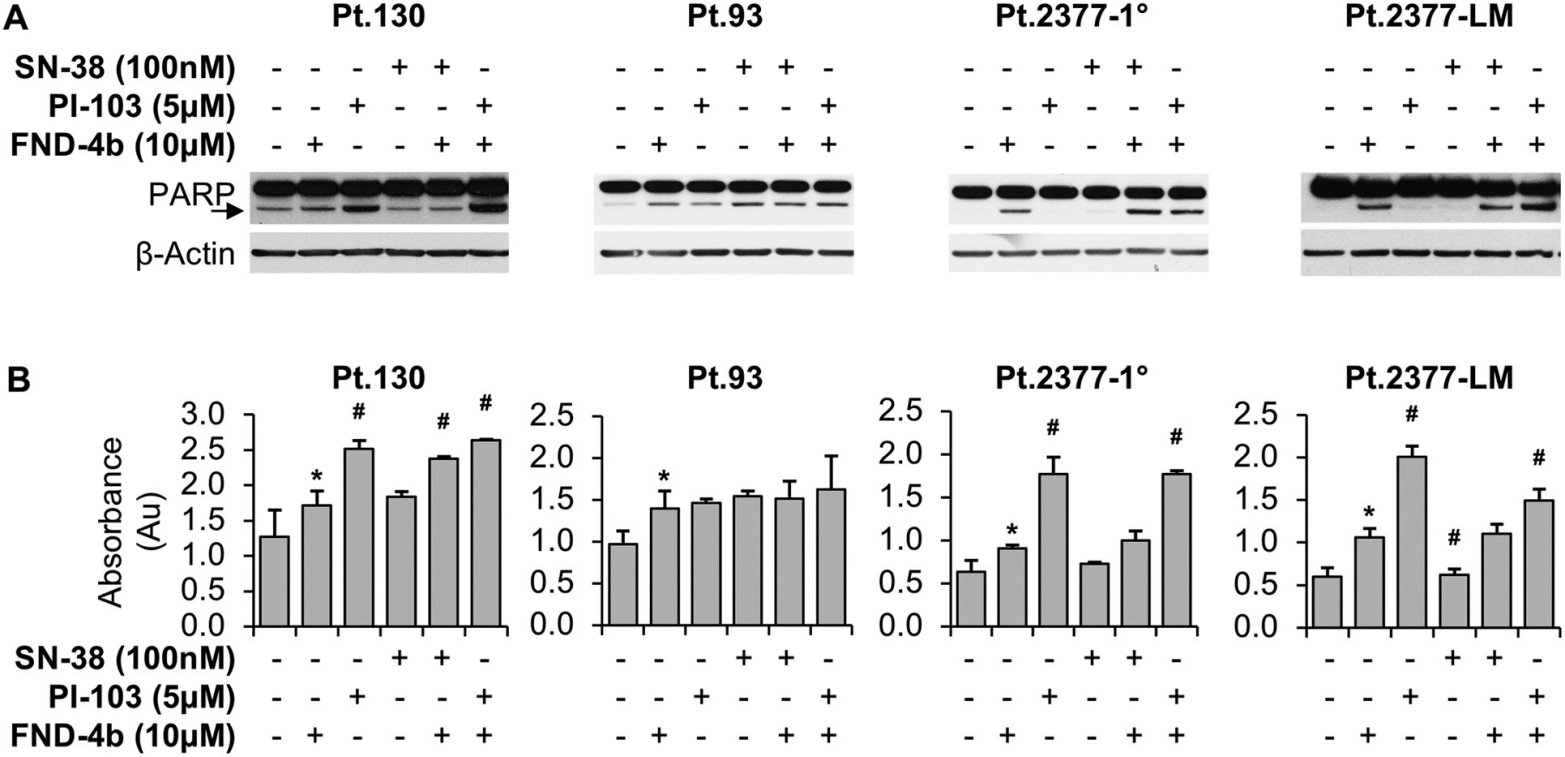
Apoptosis of PDX-derived CRC cell lines treatment combinations. (A) Western blot analysis of PDX-derived CRC cell lines treated with 10μM FND-4b, 5μM PI-103, and 100nM SN-38, alone and in combination versus untreated control (media alone) for 24h duration. PARP cleavage is indicated by an arrow; β-actin was used as a loading control. The images are representative of three independent experiments. In all CRC cell lines, PARP cleavage was increased as a result of FND-4b treatment compared to untreated control. (B) DNA fragmentation measured by Cell Death ELISA Assays of PDX-derived CRC cell lines treated with 10μM FND-4b, 5μM PI-103, and 100nM SN-38, alone and in combination for 48h duration. Graphic representations are the mean ± SD of DNA fragmentation plotted as absorbance (Au); each measurement was performed with 3 replicates. *p<0.05 vs. control and #p<0.01 vs. FND-4b alone.

To evaluate the impact of FND-4b treatment on apoptosis of the PDX-derived CRC cell lines, we first probed western blots for cleaved PARP. In all cell lines, PARP cleavage was increased following treatment with FND-4b compared with the untreated control (Fig 6A). In Pt.130 and Pt.2377-LM cell lines, the greatest increase of PARP cleavage was observed following dual therapy with FND-4b + PI-103. Alternatively, in the Pt.2377-1° cell line, PARP cleavage was greatest following treatment with the combination of FND-4b and SN-38. In Pt.93, there was no visible difference in PARP cleavage following FND-4b monotherapy compared to combination treatment.

To better delineate the effect on cell death, PDX-derived CRC cell lines were treated with combinations of FND-4b, PI-103, and SN-38 for 144h and DNA-fragments were quantitated using a Cell Death Detection ELISA. All cell lines demonstrated increased DNA fragmentation when treated with FND-4b compared to untreated control (Pt.130 p = 0.04, Pt.93 p = 0.02, Pt.2377-1° p = 0.02, Pt.2377-LM p<0.01) (Fig 6B). Further, the combination of PI-103 with FND-4b significantly increased apoptosis in Pt.130, Pt.2377-1°, and Pt.2377-LM, compared to FND-4b monotherapy (p = 0.0002, p<0.0001, and p = 0.01, respectively). In Pt.93, the apoptotic effect was similar among all treatment combinations, suggesting that this cell line is equally sensitive to all of the drugs studied. This conclusion is also supported by the results of the CytoScan SRB Cytotoxicity assay, which showed a universal decrease in cell proliferation in Pt.93 cells following treatment with any of the 3 drugs. Overall, it appears that PI-103 had the most pronounced effect on apoptosis in PDX-derived CRC cell lines, as the addition of this drug to FND-4b greatly increased DNA fragmentation (Fig 6B).

## Discussion

FND-4b represents an AMPK activator that arrests cell cycle and induces apoptosis in colon cancer cells at micromolar concentrations. Previously, we reported the ability of FND-4b to inhibit growth in CRC stem cell lines at significantly lower doses than were required by metformin treatment [10, 13]. In our current study, we evaluated the effect of FND-4b on commercially-available CRC cell lines, and extended our study to also include four PDX-derived CRC cell lines. In all CRC cell lines studied, we showed that FND-4b not only inhibited cell cycle progression, but also induced apoptosis. Moreover, the cytostatic and cytotoxic impact of FND-4b on cancer cells occurred independent of the mutations present in the CRC cell line. In addition, we studied the impact of dual FND-4b therapy on CRC growth in order to provide a comparison of the effectiveness of our novel AMPK activator to other compounds of public interest. We demonstrated that the addition of a PI3K/mTOR inhibitor (PI-103) or a DNA topoisomerase inhibitor (SN-38) to FND-4b treatment enhanced the apoptotic effect compared to treatment with FND-4b alone. Therefore, our results indicate that FND-4b is effective against CRC cells when given as a single agent and remains effective when used in combination therapy.

AMPK plays a major role in the regulation of cell metabolism and growth in both normal and neoplastic tissues [5, 6]. AMPK deficiencies have been shown to enhance cell growth and proliferation and promote tumorigenesis [5]. Simultaneously, upregulation of AMPK serves to halt the cell cycle and suppress tumor growth via inhibition of the mTOR signaling pathway [21]. High doses of AMPK activators, such as metformin and AICAR, have been shown to achieve anti-cancer effects, but the dosages required to achieve an anti-cancer effect also increases the likelihood of unwanted systemic side effects [22–26]. More recently, research has focused on developing AMPK activators that are effective at low concentrations. For example, Chen *et al*. [27] found that a novel direct-AMPK agonist, D561-0775, caused cell cycle arrest in Gefitinib-resistant non-small cell lung cancer cells at a concentration of 20μM. Law *et al*. [28] demonstrated that another novel AMPK activator, thalidezine, showed cytotoxic effects toward lung cancer, breast cancer, and liver cancer cells at treatment concentrations of less than 10μM, but in contrast, low cytotoxicity on normal liver hepatocytes was demonstrated with concentrations as high as 88μM. While these agents have not yet been tested in colon cancer, Valtorta *et al*. [29] has shown that 1,4-diaryl-2-azetidinone, a novel AMPK activator, inhibited proliferation in select colon cancer cell lines at nanomolar concentrations, but did not alter cell proliferation in other colon cancer cell lines even when the drug concentration was increased significantly, thus limiting the application of this drug. Similar to these agents, FND-4b treatment is effective at micromolar concentrations. More importantly, in contrast to the novel AMPK activators described above, in all CRC cell lines tested (regardless of mutation profile), FND-4b treatment consistently results in cell cycle arrest and induction of apoptosis. These studies provide further evidence that targeting cancer cells with novel AMPK activators represents an effective strategy with both cytostatic and cytotoxic implications.

We selected PI-103 as a comparison compound in order to include a drug that targets molecules surrounding AMPK, specifically PI3K and mTOR. Given that FND-4b activates AMPK, which in turn inhibits mTOR signaling, the upstream peptides in the mTOR signaling pathway, PI3K and AKT, would accumulate and potentially eventually override the FND-4b effect on AMPK, ultimately allowing the re-initiation of cell proliferation. We found that although FND-4b treatment did cause an accumulation of upstream pAKT, this did not have a negative impact on the ability to suppress cancer cell growth or enable apoptosis. We also showed that a dual drug regimen of FND-4b with PI-103 achieved greater pro-apoptotic impact and inhibited cell proliferation more significantly than the dual therapy of FND-4b with SN-38, or any mono drug therapy. Importantly, these effects were also noted in cell lines with known PI3K mutations, which lacked cytostatic activity when treated by PI-103 monotherapy. We speculate that the robust impact of dual FND-4 + PI-103 therapy on cell cycle inhibition is related to the target of FND-4b being downstream from the target of PI-103, as such FND-4b is not impacted by a PI3K mutation. Our findings align with those of another group that studied dual therapy of a PI3K-inhibitor (LY294002 10μM) and an AMPK activator (metformin 10mM) in ovarian cancer cells; however, in contrast, we were able to demonstrate cell cycle arrest and induction of apoptosis in CRC cells using a significantly lower dosage of FND-4b [30]. To our knowledge, no other studies have examined the combination treatment of a PI3K inhibitor and AMPK activator in CRC. However, PI-103 has been studied in combination with a number of other treatment agents for cancers such as triple-negative breast cancer, non-small cell lung cancer, and lymphoma [31–33]. Similar to our findings, these studies demonstrate that the addition of PI-103 to a drug that inhibits an alternate signaling molecule enhances the effectiveness of cell cycle inhibition.

We studied SN-38 as the second comparison treatment compound in order to compare the pro-apoptotic effects of FND-4b to an agent that functions like irinotecan, a Category 1A recommended systemic chemotherapy agent used to treat advanced or metastatic colon cancer but with a lower side effect profile than irinotecan [34–37]. We demonstrated that FND-4b treatment produces similar pro-apoptotic effects as SN-38 and has superior inhibitory effect on cell proliferation compared to SN-38. As well, the addition of SN-38 to FND-4b only increased DNA fragmentation in three cell lines studied (HCT116, LS174T, Pt.130), which we speculate is potentially attributable to APC mutations in the other cell lines (HT29, DLD1, Pt.93, Pt.2377-1°, Pt.2377-LM). To our knowledge, no other studies have examined the combination treatment of a DNA topoisomerase inhibitor and AMPK activator in colon cancer. However, numerous studies have demonstrated increased tumor cytotoxicity through a synergistic effect of SN-38 (or irinotecan) in combination with novel PARP inhibitors or oxaliplatin [38, 39]. While these studies demonstrate that the combination treatment improved cytotoxicity, the results of our study indicate that the addition of SN-38 to FND-4b did not consistently improve cytotoxicity compared to treatment with FND-4b alone.

In summary, we have shown that despite a variety of mutation profiles in the studied CRC cell lines, treatment with FND-4b consistently results in cell cycle arrest and apoptosis. Furthermore, these results are noted with treatment of FND-4b at micromolar concentrations, much lower concentrations than have been shown effective for other drugs, such as metformin. Importantly, we demonstrate that FND-4b is a novel and effective inhibitor of CRC growth either alone or in combination with PI-103 and SN-38. Given its efficacy at micromolar concentrations, FND-4b should be the focus of future CRC treatment studies—particularly those that evaluate its efficacy in an in vivo model.

## Supporting information

S1 Table Compounds of interest. (DOCX)

S2 Table Key genetic mutation profile of cell lines studied. (X) Denotes a damaging mutation. Six of the cell lines studied have potentially damaging PI3K mutations: HCT116, HT29, LS174T, DLD1, Pt.2377-1°, and Pt.2377-LM.
(DOCX)

S3 Table Detailed profile of key genetic mutation in cell lines studied. (DOCX)
