# Supplementary material for: Novel chemotherapeutic agent, FND-4b, activates AMPK and inhibits colorectal cancer cell proliferation
Source: PLoS One. 2019 Oct 24;14(10):e0224253. doi: 10.1371/journal.pone.0224253 (PMC6812860; doi:10.1371/journal.pone.0224253)
Supplement: S1 Table — (DOCX) [file pone.0224253.s001.docx]

**Supplemental Table 1. Compounds of Interest.**

| **Compound** | **Formula** | **Molecular Weight (kDa)** | **Chemical Structure** |
| --- | --- | --- | --- |
| **FND-4b** | C_15_H_9_ClF_6_N_2_O_2_S | 430.749 |  |
| **PI-103** | C_19_H_16_N_4_O_3_ | 348.36 | 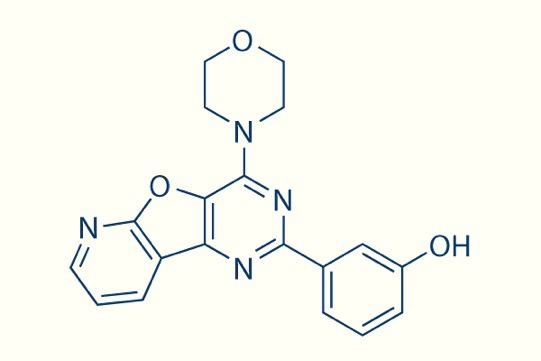 |
| **SN-38** | C_22_H_20_N_2_O_5_ | 392.4 | HO  O  O  N  O  N  HO |
