# Supplementary material for: Novel chemotherapeutic agent, FND-4b, activates AMPK and inhibits colorectal cancer cell proliferation
Source: PLoS One. 2019 Oct 24;14(10):e0224253. doi: 10.1371/journal.pone.0224253 (PMC6812860; doi:10.1371/journal.pone.0224253)
Supplement: S2 Table — (X) Denotes a damaging mutation. Six of the cell lines studied have potentially damaging PI3K mutations: HCT116, HT29, LS174T, DLD1, Pt.2377-1°, and Pt.2377-LM. (DOCX) [file pone.0224253.s002.docx]

**Supplemental Table 2. Key Genetic Mutation Profile of Cell Lines Studied.** (x) Denotes a damaging mutation. Six of the cell lines studied have potentially damaging PI3K mutations: HCT116, HT29, LS174T, DLD1, Pt.2377-1⁰, and Pt.2377-LM.

|  | APC | BRAF | FGFR1 | KRAS | mTOR | PI3K | TP53 |
| --- | --- | --- | --- | --- | --- | --- | --- |
| **HCT116** |  |  |  | **x** |  | **x** |  |
| **HT29** | **x** | **x** |  |  |  | **x** | **x** |
| **LS174T** |  |  |  | **x** |  | **x** |  |
| **DLD1** | **x** |  |  | **x** |  | **x** | **x** |
| Pt.93 | x | x |  |  | x |  |  |
| Pt.130 |  | x | x |  |  |  | x |
| **Pt.2377-1⁰** | **x** |  |  | **x** |  | **x** |  |
| **Pt.2377-LM** | **x** |  |  | **x** |  | **x** |  |
