# Supplementary material for: Novel chemotherapeutic agent, FND-4b, activates AMPK and inhibits colorectal cancer cell proliferation
Source: PLoS One. 2019 Oct 24;14(10):e0224253. doi: 10.1371/journal.pone.0224253 (PMC6812860; doi:10.1371/journal.pone.0224253)
Supplement: S3 Table — (DOCX) [file pone.0224253.s003.docx]

**Supplemental Table 3. Detailed Profile of Key Genetic Mutation in Cell Lines Studied.**

| **Human**  **Specimen**  **ID** | **Primary**  **Staging** | **Age** &  **Sex** | **Pathology** | **Mutation**  **Type** | **Gene** | **Gene**  **ID** | **cDNA Change** | **Protein Change** |
| --- | --- | --- | --- | --- | --- | --- | --- | --- |
| Pt93 | T3N1bM1 | 63M | Metastatic peritoneum & abdominal wall poorly differentiated adenocarcinoma from colon primary | Frameshift | APC | 324 | NM_000038.5:c.5375dupA | NP_000029.2:p.Asn1792LysfsTer7 |
|  |  |  |  | Missense | BRAF | 673 | NM_004333.4:c.1799T>A | NP_004324.2:p.Val600Glu |
|  |  |  |  | Missense | MTOR | 2475 | NM_004958.3:c.6709A>G | NP_004949.1:p.Ile2237Val |
| Pt130 | T3N0M1a | 76M | Metastatic colonic adenocarcinoma | Missense | BRAF | 673 | NM_004333.4:c.1799T>A | NP_004324.2:p.Val600Glu |
|  |  |  |  | Missense | FGFR1 | 2260 | NM_001174067.1:c.2557C>T | NP_001167538.1:p.Arg853Cys |
|  |  |  |  | Frameshift | TP53 | 7157 | NM_000546.5:c.455delC | NP_000537.3:p.Pro152ArgfsTer18 |
|  |  |  |  | Frameshift | TP53 | 7157 | NM_000546.5:c.267dupC | NP_000537.3:p.Ser90LeufsTer59 |
| Pt2377-1⁰ | T3N0M1a | 66F | Metastatic colonic adenocarcinoma (primary tumor) | Stop gained | APC | 324 | NM_000038.5:c.2212A>T | NP_000029.2:p.Lys738Ter |
|  |  |  |  | Frameshift | APC | 324 | NM_000038.5:c.4666dupA | NP_000029.2:p.Thr1556AsnfsTer3 |
|  |  |  |  | Missense | KRAS | 3845 | NM_033360.2:c.436G>A | NP_203524.1:p.Ala146Thr |
|  |  |  |  | Missense | PIK3CA | 5290 | NM_006218.2:c.1633G>A | NP_006209.2:p.Glu545Lys |
| Pt2377-LM | T3N0M1a | 66F | Metastatic colonic adenocarcinoma (Liver metastasis) | Stop gained | APC | 324 | NM_000038.5:c.2212A>T | NP_000029.2:p.Lys738Ter |
|  |  |  |  | Frameshift | APC | 324 | NM_000038.5:c.4666dupA | NP_000029.2:p.Thr1556AsnfsTer3 |
|  |  |  |  | Missense | KRAS | 3845 | NM_033360.2:c.436G>A | NP_203524.1:p.Ala146Thr |
|  |  |  |  | Missense | PIK3CA | 5290 | NM_006218.2:c.1633G>A | NP_006209.2:p.Glu545Lys |
| HCT116  CCL-247™ | Dukes’  type D | 48M | Colon adenocarcinoma | Missense | KRAS | 3845 | NM_033360.2:c.38G>A | p.Gly13Asp |
|  |  |  |  | Missense | PIK3CA | 5286 | NM_006218.2: c.3140A>G | p.His1047Arg |
| HT29  HTB-38™ | Dukes’  type C | 44F | Colon adenocarcinoma | Frameshift | APC | 324 | NM_000038.5:c.4666_4667insA | p.Thr1556fs*3 |
|  |  |  |  | Substitution–  Nonsense | APC | 324 | NM_000038.5:c.2557G>T | p.Glu853* |
|  |  |  |  | Missense | BRAF | 673 | NM_004333.4:c.1799T>A | p.Val600Glu |
|  |  |  |  | Missense | BRAF | 673 | NM_004333.4:c.356C>G | p.Thr119Ser |
|  |  |  |  | Missense | PIK3CA | 5286 | NM_006218.2:c.1345C>A | p.Pro449Thr |
|  |  |  |  | Missense | TP53 | 7157 | NM_000546.5:c.818G>A | p.Arg273His |
| LS174T  CL-188™ | Dukes’  type B | 58F | Colon adenocarcinoma | Missense | KRAS | 3845 | NM_033360.2:c.35G>A | p.Gly12Asp |
|  |  |  |  | Missense | PIK3CA | 5286 | NM_006218.2:c.3140A>G | p.His1047Arg |
| DLD1  CCL-221^TM^ | Dukes’ type C | Adult M | Colon adenocarcinoma | Missense | APC | 324 | NM_000038.5:c.2180G>T | p.Arg727Met |
|  |  |  |  | Missense | APC | 324 | NM_000038.5:c.2979G>T | p.Lys993Asn |
|  |  |  |  | Frameshift | APC | 324 | NM_000038.5:c.4248delC | p.Ile1417fs*2 |
|  |  |  |  | Missense | APC | 324 | NM_000038.5:c.6496C>T | p.Arg2166Ter |
|  |  |  |  | Missense | KRAS | 3845 | NM_033360.2:c.38G>A | p.Gly13Asp |
|  |  |  |  | Missense | PIK3CA | 5286 | NM_006218.2:c.1633G>A | p.Glu545Lys |
|  |  |  |  | Missense | PIK3CA | 5286 | NM_006218.2:c.1645G>A | p.Asp549Asn |
|  |  |  |  | Missense | TP53 | 7157 | NM_000546.5:c.722C>T | p.Ser241Phe |
